# Supplementary material for: Synthesis of New Cobalt(III) Meso-Porphyrin Complex, Photochemical, X-ray Diffraction, and Electrical Properties for Photovoltaic Cells
Source: Molecules. 2022 Dec 13;27(24):8866. doi: 10.3390/molecules27248866 (PMC9785790; doi:10.3390/molecules27248866)
Supplement: Supplementary file 1 [file molecules-27-08866-s001.zip › molecules-2041113-supplementary.pdf]

Synthesis of New Cobalt(III) *Meso*-Porphyrin Complex. Photochemical, X-ray diffraction and Electrical Properties for Photovoltaic Cells

Soumaya Nasri [1,2] *, Mouhieddinne Guergueb [2], Jihed Brahmi [2], Youssef O. Al-Ghamdi [1], Florian Molton [3], Frédérique Loiseau[3] , Ilona Turowska-Tyrk [4] and Habib Nasri [1]

[1] Department of Chemistry, College of Science Al-Zulfi, Majmaah University, Majmaah 11952, Saudi Arabia
[2] Laboratory of Physical Chemistry of Materials, Faculty of Sciences of Monastir, University of Monastir, Ave-nue de L'environnement, Monastir 5019, Tunisia
[3] Département de Chimie Moléculaire, 301 rue de la Chimie, Université Grenoble Alpes, CS 40700, CEDEX 9, 38058 Grenoble, France
[4] Faculty of Chemistry, Wrocław University of Science and Technology, 27 Wybrzeże Wyspiańskiego, 50-370 Wrocław, Poland
* Correspondence: soumaya.n@mu.edu.sa

Contents

1. **Experimental.. 2**
2. **X-ray Molecular structure 3**
3. **IR spectroscopy… 7**
4. **^{1}H NMR 9**
5. **UV/Vis Spectroscopy… 10**
6. **Photodegradation 11**

1. Experimental

1.1. Elemental analysis, UV/Vis, IR and ^{1}H NMR spectrometry

Elementary analyses were performed within the ICMG Chemistry Nanobio Platform, Grenoble. The UV/Vis spectra were recorded with a WinASPECT PLUS (validation for SPECORD PLUS version 4.2) scanning spectrophotometer. Fourier-transformed IR spectra were recorded on a PerkinElmer Spectrum Two FT-IR spectrometer and the ^{1}H NMR spectra were obtained at room temperature on a Bruker 300 Ultra shield spectrometer.

1.2. Fluorescence spectroscopy

A Fluoromax-4 spectrofluorometer was used to obtain emission spectra in dichloromethane at roomtemperature. In addition, the quantum efficiency of emissions was examined in dichloromethaneusing the optical method **[25]**. The [Zn(TPP)] solution was used as the quantum yield standard (Φ =0.031). Luminescence lifetime revelation was realized after irradiation at λ of 430 nm. The luminescence decay was analyzed by Picoquant FLUOFIT software.

1.3. X-ray diffraction analysis of complex 4

Data collection for compound **4** was carried out using a Xcalibur, Onyx, Nova diffractometer equipped with a CuKα radiation source (λ= 1.54056 Å). Data measurements were made at room temperature. The reflections were rescaled and adjusted for absorption effects by use of CrysAlis program **[26]**. The structure was determined by direct methods using the SIR-2004 software **[27]** andwere refined by the least squares matrix methods on F^2 through the SHELXL-2014 software **[28]**. The CO_2 group of the nicotinoyl chloride (NTC) axial ligand is disordered in two positions (C50A-O1A-Cl6A and C50B-O1B-Cl6B) with an occupancy factor of the major position of 0.616 (0.008) (Figure S1). The dichlorobenzene solvent molecule is also disordered in two positions (C51A-Cl7A-Cl8A and C51B-Cl7B-Cl8B) with refine occupancy factors of 0.883 (0.004) and 0.117 (0.004), respectively (Figure S1). The anisotropic displacement ellipsoids of the disordered atoms of the NTC axial ligandand the dichloromethane solvent molecules are very elongated, which indicate that

they are statistically disordered. Therefore, the PLAT and SIMU constraint commands were used. To correctthe geometry of these two disordered moieties, we used the DANG and the DFIX constraint commands.

The hydrogen atoms were placed in calculated positions and treated as riding on their parent atoms. The geometrical calculations were made using the program PLATON **[29]** and the moleculardrawings and the packing diagrams were performed using the software MERCURY **[30]**. The

crystallographic data and structural refinement details of **4** are presented in Table S1. Selected bondlengths and angles for the compound are shown in Table S2.
The crystal data were deposed in the Cambridge Crystallographic Data base with the CCDCdeposition number 2143744.

1.4. Hirshfeld surface analysis

Hirshfeld surface scanning and the related fingerprint information of complex **4** were calculatedusing the Crystal Explorer17 software **[31]**.

2. X-Ray Molecular structure

Table S1. Crystal data and structural refinement for $[Co^{III}(TClPP)(NTC)]{\bullet}CH_2Cl_2$ (**4**).

Formula	$C_{51}H_{30}CoN_5Cl_8O$
Crystal System	monoclinic
Crystal	$P2_1/c$
a (Å)	12.5872(2)
b (Å)	15.2540(2)
c (Å)	24.526(3)
β (°)	96.827()
V(Å^3)	4675.75(11)
Z	4
$\rho_{calc.}$/ g cm^{-3}	1.522
μ/ mm^{-1}	7.451
$F(000)$	2168
Crystal size (mm^3)	0.41 x 0.31 x 0.25
Crystal Color	purple
Crystal Shape	prism
T(K)	293 (2)
$Cu\ K\alpha$ radiation	λ = 1.54056 Å
◎$_{min}$ - ◎$_{max}$ (°)	3.416– 67.680
Limiting indices	-15◎ h ◎14, -19◎ k ◎19, -30◎ l ◎30
R(int)	0.0397
Reflections collected/unique	108274 / 9566
Observed data [$I_o > 2\sigma(F_o)$]	8022
Parameters/Rest	647 /202
S [Goodness of fit, all data]	1.101
$R_1{}^a$, wR^b [$F_o > 4\sigma(F_o)$]	R_1 = 0.0617, wR_2 = 0.1665
$wR_2{}^b$ [all data]	R_1 = 0.0716, wR_2 = 0.1774
Min./max. res. (eÅ^{-3})	0.991 / -0.525
CCDC	2143744

^{a} :$R1 = \Sigma||F_O|-|F_C||/\Sigma|F_O|$, ^{b} :$wR2 = \{\Sigma[w(|F_O|^2-|F_C|^2)^2]/\Sigma[w(|F_O|^2)^2]\}^{1/2}$.

Table S2. Selected bond lengths (Å) and angles (°) of **4**.

Cobalt(III) coordination polyhedron			
Co1◎N1	1.943(3)	N2-Co1-N4	179.08(12)
Co1-N2	1.942(3)	N3-Co1-N4	89.74(12)
Co1-N3	1.948(3)	N5-Co1-N1	90.14(12)
Co1-N4	1.955(3)	N5-Co1-N2	88.43(9)
Co1-N5	1.997(3)	N5-Co1-N3	88.63 12)
Co1-Cl5	2.2282(10)	N5-Co1-N4	89.60(12)
N1-Co1-N2	89.67(12)	Cl5-Co1-N1	90.68(9)
N1-Co1-N3	178.67(12)	Cl5-Co1-N2	88.43(9)
N1-Co1-N4	89.73(12)	Cl5-Co1-N3	90.56(9)
N2-Co1-N3	90.85(12)	Cl5-Co1-N4	92.28(9)
		Cl5-Co1-N5	177.95(9)
Nicotinoyl chloride (NTC)Axial ligand			
N5-C45	1.323(5)	C50B-Cl6B	1.662(8)
C45-C46	1.383(6)	Co-N5-C45	120.4(3)
C46-C47	1.372(7)	Co-N5-C49	121.5(3)
C47-C48	1.358(7)	C45-N5-C49	118.0(3)
C48-C49	1.376(6)	C46-C50A-O1A	121.6(8)
C49-N5	1.348(5)	C46-C50B-O1B	127.6(9)
C46-C50A	1.484(7)	C46-C50A-Cl6A	127.1(7)
C46-C50B	1.478(9)	C46-C50B-Cl6B	108.8(8)
C50A-O1A	1.189(8)	O1A-C50A-Cl6A	112.1(3)
C50A-Cl6A	1.697(7)	O1B-C50B-Cl6B	112.2(10)
C50B-O1B	1.237(9)		

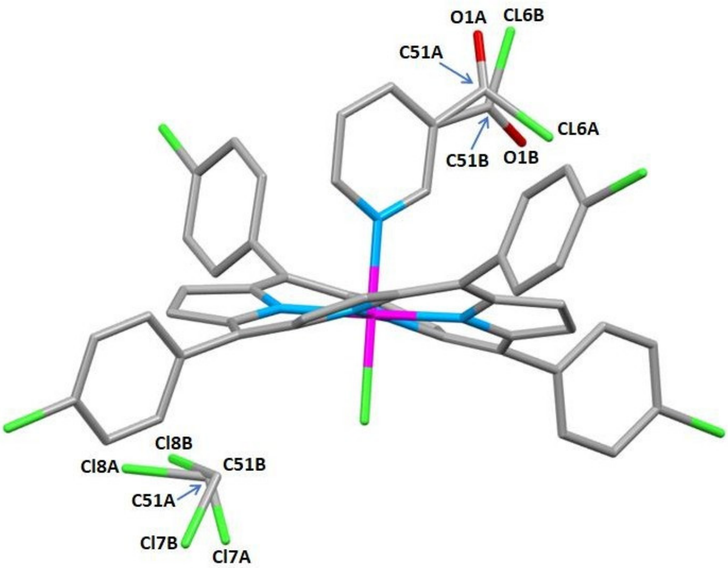

Figure S1. Representation de la structure du complex [Co^{III}(TClPP)Cl(NTC)]•CH_2Cl_2 (**4**) showing the disordered NTC axial ligand and the disordered dichloromethane solvent molecule.

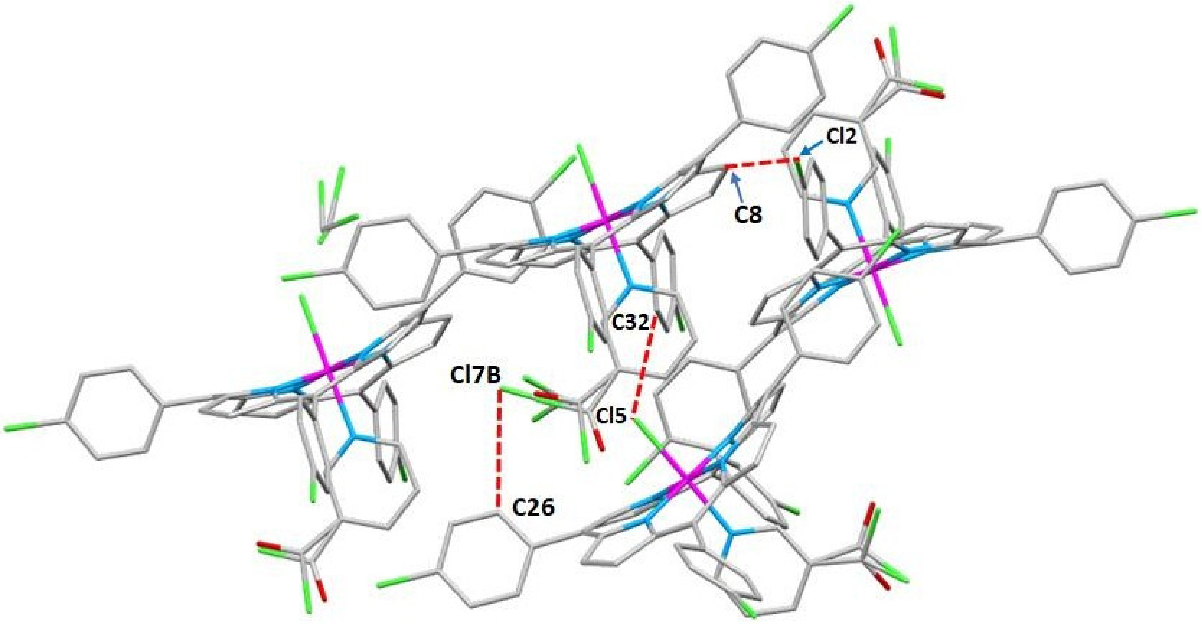

Figure S2. Schematic representation showing the weak C8–H8··Cl2 intermolecular interaction in the crystallattice if **4**.

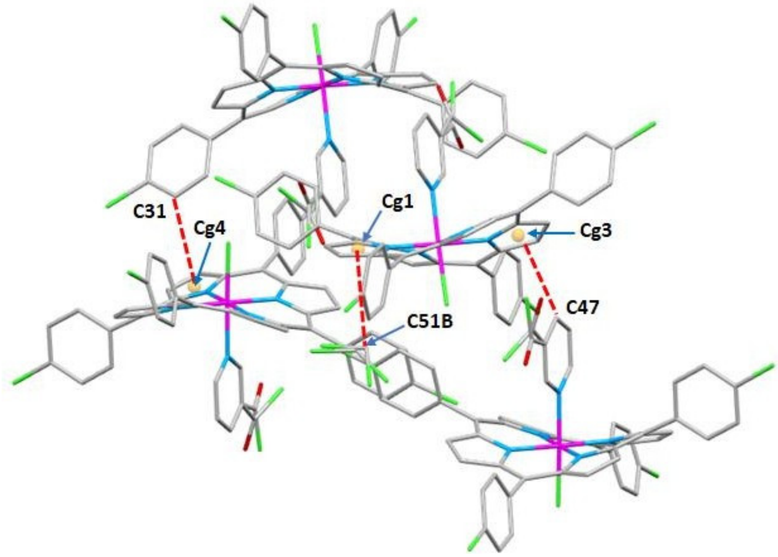

Figure S3. Schematic representation showing the weak C–H··Cl —H···Cg intermolecular interactions in
and Cthe crystal lattice if **4**.

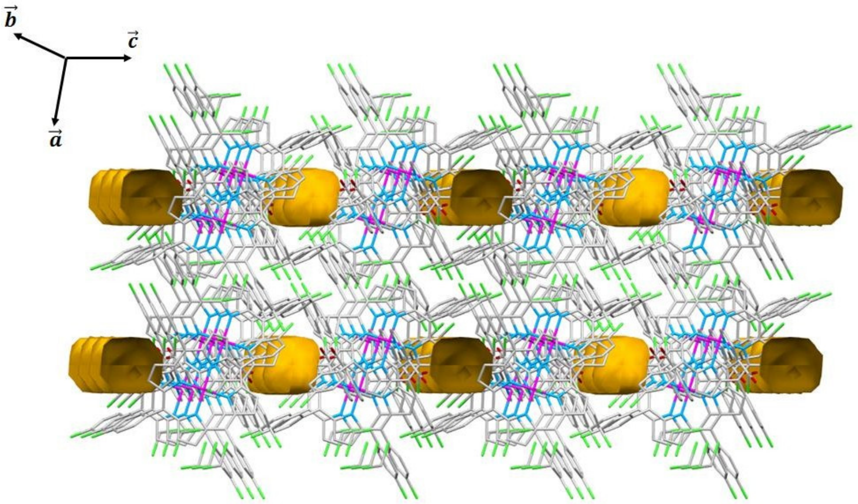

Figure S4. Packing diagram of compound **4** showing voids calculated for a ball radius of 1.2 Å and a grid of 0.7Å.

Table S3. Selected intermolecular interactions for compound**4**.

D –H···A[a]	Symmetry of A	D···A (Å)	D–H···A (°)
C8-H8…Cl2	2-x,-y,-z	3.398(4)	137
C32-H32…Cl5	2-x,-1/2+y,1/2-z	3.374 (3)	133

C26-H26…Cl7B	x,y,z	3.644(11)	153

C31-H31…Cg4	2-x,-1/2+y,1/2-z	3.503(3)	126
C47-H47…Cg3	2-x,-1/2+y,1/2-z	3.458(5)	132
C51A-H51B-Cg1	x,y,z	3.675(6)	152
C51B-H51C-Cg1	x,y,z	3.61(5)	136

[a]: D = donor atom and A = acceptor atom.

Cg1 is the centroid of the N1/C2-C4 pyrrole ring. Cg3 is the centroid of the N3/C11-C14 pyrrole ring.Cg4 is the centroid of the N4/C16-C19 pyrrole ring.

3. IR spectroscopy

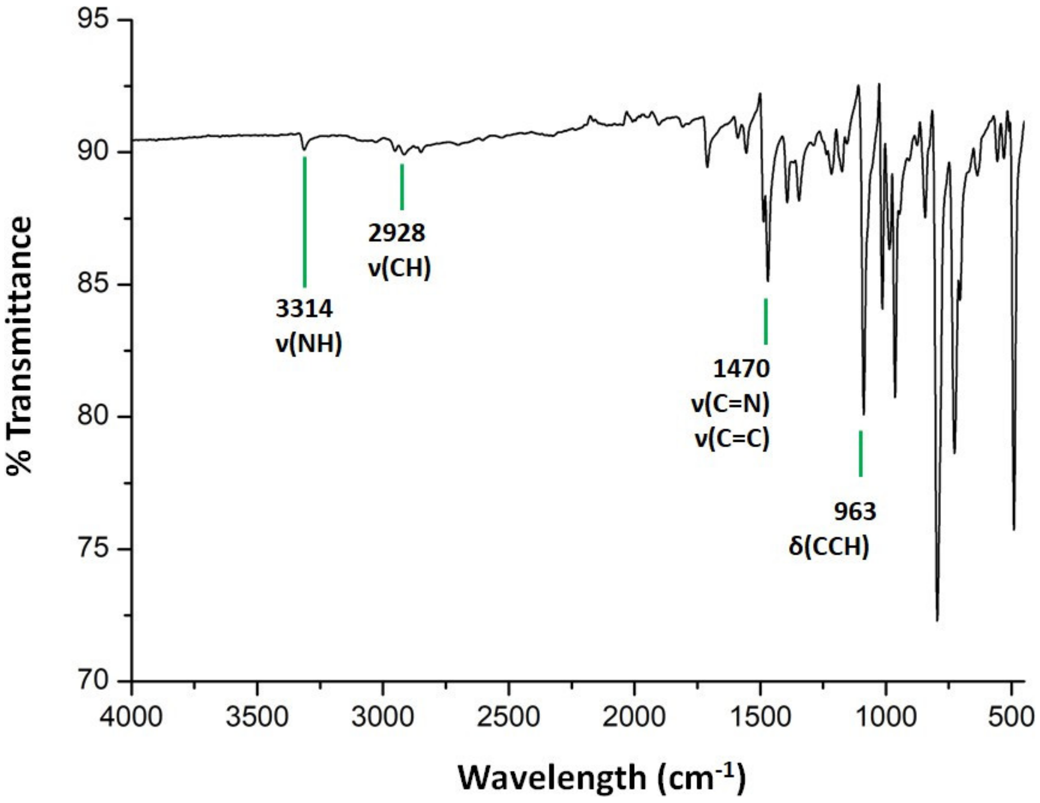

Figure S5. IR spectrum of the free base porphyrin (H_2TClPP) (**1**).

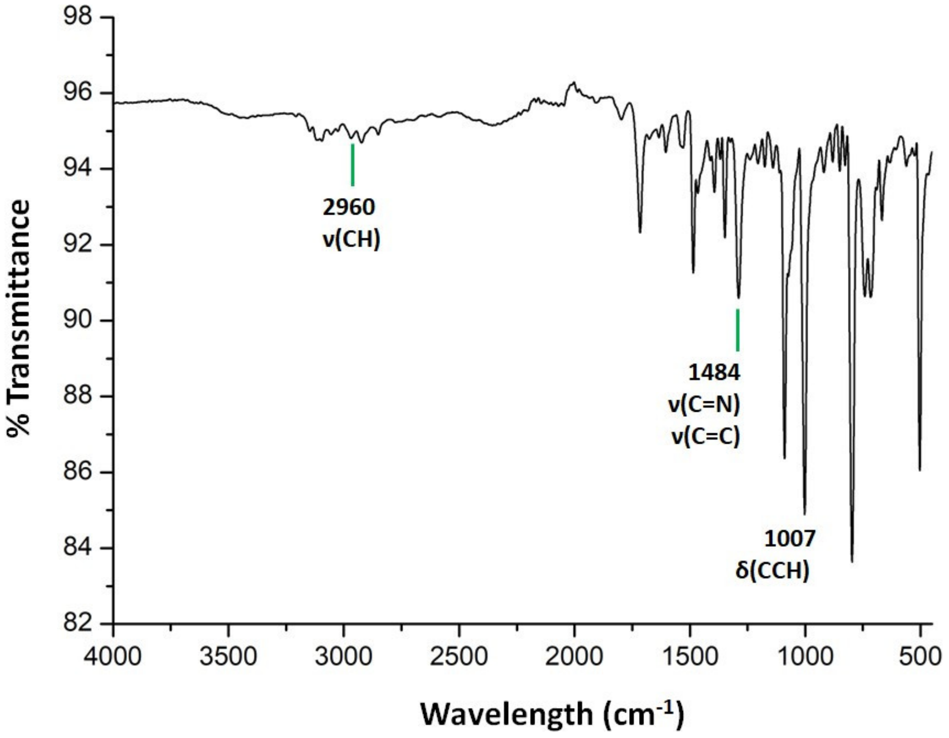

Figure S6. IR spectra of the complex [Co^{II}(TClPP)] (**2**).

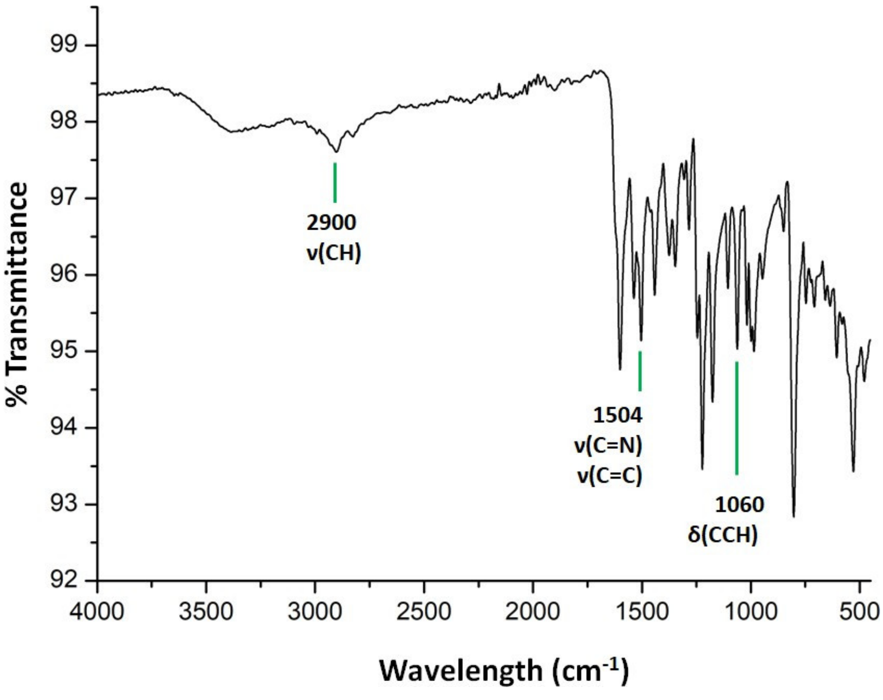

Figure S7. IR spectra of the complex [Co^{III}(TClPP)Cl] (**3**).

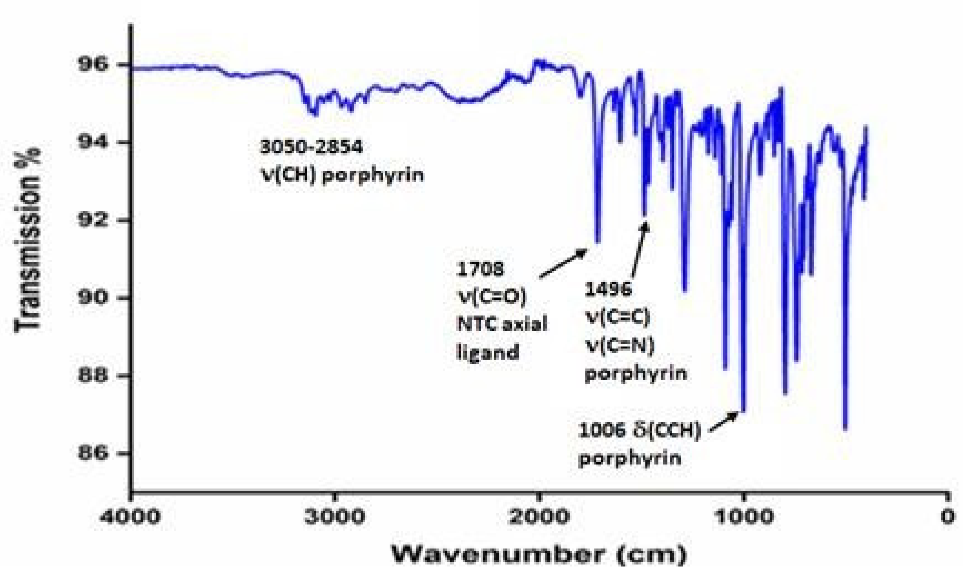

Figure S8. IR spectrum (solid state) of [Co^{III}(TClPP)Cl(NTC)]·CH_2Cl_2 (**4**).

4. ^{1}H NMR spectroscopy

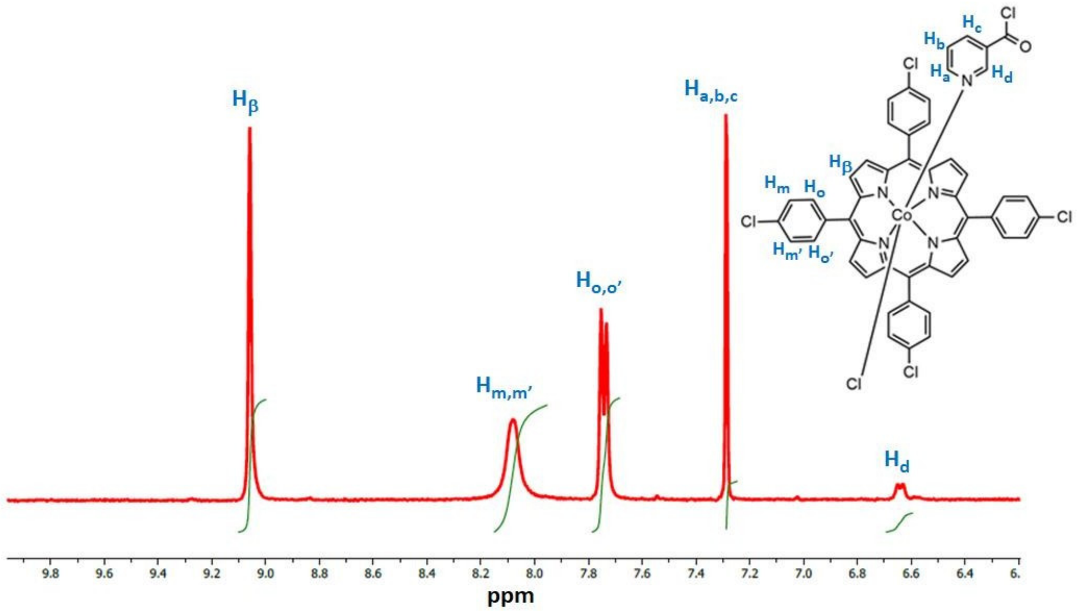

Figure S9. ^{1}H NMR spectra of **4** recorder in $CDCl_3$ with a concentration ~ 10^{-3} M.

5. UV/Vis Spectroscopy

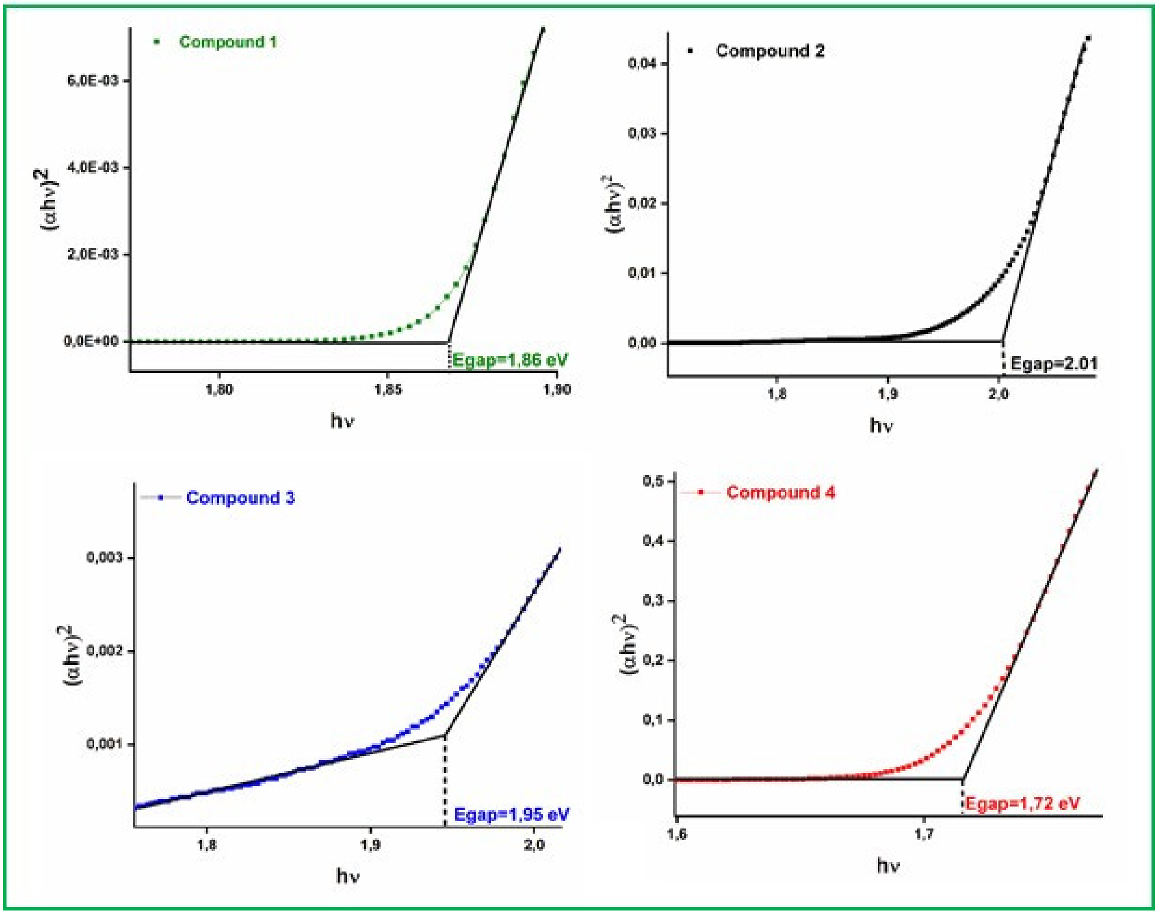

Figure S10. Curves of $(\alpha h\upsilon)^2$ against photon energy *E* of compounds **1-4**.

6. Photodegradation

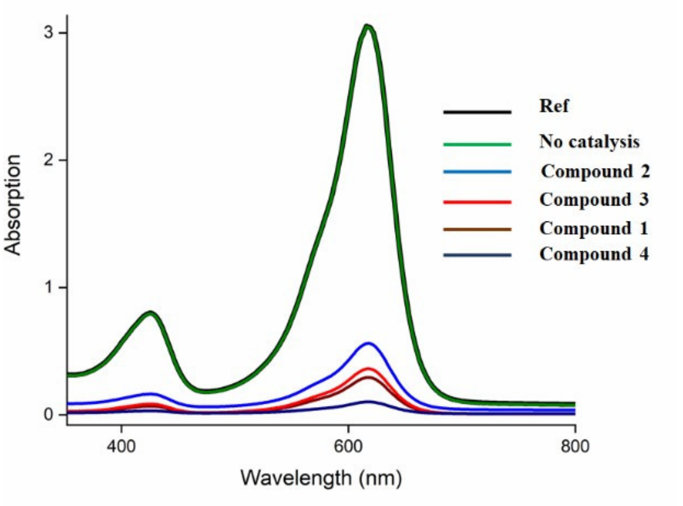

Figure S11. Variation of the ◎$_{max}$ values of the absorption bands of Malachite green (MG) dye in thepresence of **1-4** (5 mg). The concentration of MG is 20 mg.L^{-1} and pH = 7.

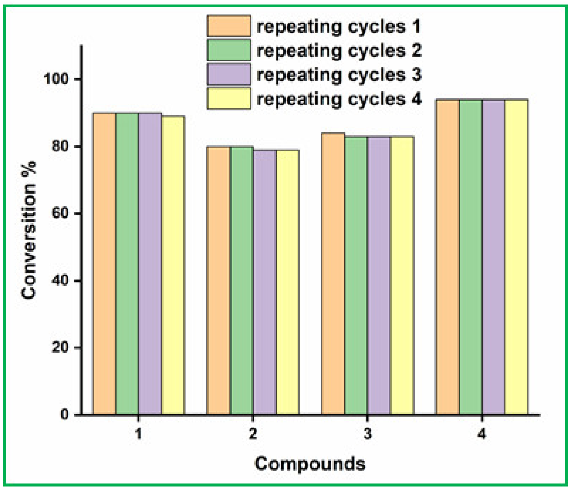

Figure S12. Circulatory experiments of photo catalytic degradation of dye using four compounds **1-4** asphoto catalysts.
